# Treatment of Thoracolumbar Pyogenic Spondylitis with Minimally Invasive Posterior Fixation without Anterior Lesion Debridement or Bone Grafting: A Multicenter Case Study

**DOI:** 10.3390/jcm12030932

**Published:** 2023-01-25

**Authors:** Hisanori Gamada, Toru Funayama, Akihiro Yamaji, Shun Okuwaki, Tomoyuki Asada, Shigeo Izawa, Hiroshi Kumagai, Kengo Fujii, Kuniaki Amano, Itsuo Shiina, Masaki Tatsumura, Masafumi Uesugi, Tsukasa Nakagawa, Masashi Yamazaki, Masao Koda

**Affiliations:** 1 Department of Orthopaedic Surgery, Faculty of Medicine, University of Tsukuba, 1-1-1 Tennodai, Tsukuba 305-8575, Japan; 2 Department of Orthopaedic Surgery, Ibaraki Western Medical Center, 555 Otsuka, Chikusei 308-0813, Japan; 3 Department of Orthopaedic Surgery, Ichihara Hospital, 3691 Osone, Tsukuba 300-3253, Japan; 4 Department of Orthopaedic Surgery, Kenpoku Medical Center, Takahagi Kyodo Hospital, 1006-9 Kamiteduna-Agehocho, Takahagi 318-0004, Japan; 5 Department of Orthopaedic Surgery, Tsukuba Central Hospital, 1589-3 Kashiwada-Cho, Ushiku 300-1211, Japan; 6 Department of Orthopaedic Surgery, Showa General Hospital, 8-1-1 Hanakonagei, Kodaira 187-0002, Japan; 7 Department of Orthopaedic Surgery, Moriya Daiichi Hospital, 1-17 Matsumaedai, Moriya 302-0102, Japan; 8 Department of Orthopaedic Surgery and Sports Medicine, Tsukuba University Hospital Mito Clinical Education and Training Center, Mito Kyodo General Hospital, 3-2-7 Miyamachi, Mito 310-0015, Japan; 9 Department of Orthopaedic Surgery, Ibaraki Seinan Medical Center Hospital, 2190 Sashima, Sakai 306-0433, Japan

**Keywords:** pyogenic spondylitis, instrumentation, minimally invasive spine stabilization, posterior fixation

## Abstract

The usefulness of minimally invasive posterior fixation without debridement and autogenous bone grafting remains unknown. This multicenter case series aimed to determine the clinical outcomes and limitations of this method for thoracolumbar pyogenic spondylitis. Patients with thoracolumbar pyogenic spondylitis treated with minimally invasive posterior fixation alone were retrospectively evaluated at nine affiliated hospitals since April 2016. The study included 31 patients (23 men and 8 women; mean age, 73.3 years). The clinical course of the patients and requirement of additional anterior surgery constituted the study outcomes. The postoperative numerical rating scale score for lower back pain was significantly smaller than the preoperative score (5.8 vs. 3.6, *p* = 0.0055). The preoperative local kyphosis angle was 6.7°, which was corrected to 0.1° after surgery and 3.7° at the final follow-up visit. Owing to failed infection control, three patients (9.6%) required additional anterior debridement and autogenous bone grafting. Thus, in this multicenter case series, a large proportion of patients with thoracolumbar pyogenic spondylitis could be treated with minimally invasive posterior fixation alone, thereby indicating it as a treatment option for pyogenic spondylitis.

## 1. Introduction

The gold standard treatment for pyogenic spondylitis constitutes conservative treatment with antibiotics and rest, and surgical treatment is implemented in cases where conservative treatment fails [1]. Furthermore, the standard surgical treatment procedure for pyogenic spondylitis has comprised direct lesion debridement and autogenous bone grafting via the anterior approach [1]. Recently, relatively less invasive surgical procedures, such as percutaneous endoscopic debridement, percutaneous posterior intervertebral fusion, and minimally invasive anterior autograft and posterior fixation, have been developed to achieve infection control, early ambulation, and favorable outcomes [2,3,4,5,6,7]. Moreover, posterior instrumentation without debridement and autogenous bone grafting exhibits the potential for controlling infection in pyogenic spondylitis via local stabilization [8,9,10]. In addition, minimally invasive posterior fixation using only percutaneous pedicle screws (PPS) constitutes one of the less invasive surgical procedures for pyogenic spondylitis [11,12,13].

However, the efficacy of this method without the debridement of infected lesions and bone grafting remains controversial.

This study aimed to determine the clinical outcomes of the minimally invasive posterior fixation procedure for thoracolumbar pyogenic spondylitis without anterior lesion debridement and bone grafting.

## 2. Materials and Methods

This study included patients with thoracolumbar pyogenic spondylitis who had undergone minimally invasive posterior fixation without anterior lesion debridement or bone grafting. The patients were observed for at least 6 months following surgery. The study was designed as a retrospective multicenter case series, with patients examined at nine affiliated hospitals from April 2016 to March 2022. The following were the inclusion criteria: (1) patients with pyogenic spondylitis who had not responded to conservative treatment and had undergone minimally invasive posterior fixation. Herein, minimally invasive posterior fixation is defined as posterior fixation without lesion debridement and bone grafting. The conservative treatment protocol comprised bed rest and intravenous antibiotics, and the antibiotic type and dose were administered at the discretion of each hospital. Patients were considered unresponsive to conservative treatment in case of no improvement in pain or C-reactive protein concentration following 6 weeks of conservative treatment, or if the patient was unable to continue conservative treatment owing to factors such as severe lower back pain; (2) patients in whom thoracic, lumbar, and sacral vertebrae were all affected; (3) patients who did not undergo direct lesion debridement and bone grafting; (4) the surgical procedures that the patients underwent were chiefly percutaneous; and (5) whether or not the patients had a paravertebral abscess. The exclusion criteria included cases with severe neurological symptoms due to epidural abscesses or other conditions requiring early abscess drainage, and cases in which direct posterior or anterior lesion debridement and bone grafting were performed simultaneously with minimally invasive fixation.

Finally, this study included 31 patients (23 men and 8 women) who were retrospectively examined. The mean age of the patients at the time of surgery was 73.3 ± 8.2 years (mean ± SD) (range: 56–85 years). The mean preoperative conservative treatment period was 52.7 days (range: 14–185 days). The mean postoperative follow-up period was 13.7 months (range: 6–36 months). The most commonly affected vertebral site was the lumbar spine (16 cases), followed by the thoracic spine (9 cases). In 17 cases, paravertebral abscesses involving the epidural space and iliopsoas muscles were observed (Table 1).

The most common causative organisms included *Escherichia coli* and *Streptococcus* species (six cases each), and the causative organism could not be identified in 12 cases (Table 2). The causative organism was identified based on blood culture alone in 15 cases, local specimen culture alone in 2 cases, and both blood and local specimen culture in 2 cases, whereas 12 cases could not be identified. In the cases wherein the causative organism could not be identified, antibiotics were administered at the discretion of each hospital, and cephazolin and sulbactam/ampicillin were often empirically administered.

The surgery items analyzed included operation duration, intraoperative blood loss, range of fixation, and whether or not pedicle screws were inserted into the affected vertebrae. The postoperative and clinical item analyzed were the duration of postoperative intravenous antibiotics, perioperative complications, numerical rating scale (NRS) for preoperative lower back pain, time at ambulation and discharge from hospital, postoperative period for ambulation, days required to normalize the C-reactive protein concentration, local kyphosis of the affected vertebrae at preoperative/postoperative/last follow-up, recurrence of infection, and additional surgery on the affected vertebrae.

All surgeries were performed under general anesthesia in the prone position. The intraoperative correction of kyphosis was performed only in the natural prone position. No screw- or rod-controlled correction of kyphosis was performed.

Antibiotics were administered intravenously as sensitive agents following surgery till normalization of the C-reactive protein concentration, and then orally for 1–2 months. The angle between the most proximal and most distal endplates of the affected vertebrae in the lateral radiogram was used to calculate local kyphosis. After controlling the infection, recurrence was defined as the re-elevation of inflammatory markers and/or magnetic resonance imaging signal intensity change of the vertebral body, including at other levels.

### Statistical Methods

The Wilcoxon signed-rank test was employed to determine the change in NRS and local kyphosis. The significance level was *p* < 0.05. JMP^®^ 10 (SAS Inc., Cary, NC, USA) was used for statistical analysis.

## 3. Results

The mean operation duration and intraoperative blood loss were 184 min and 221 mL, respectively. The average number of fixed vertebrae was 5.9, with 6 and 7 vertebrae being the most common (eight cases each). Pedicle screws were inserted into the infected vertebrae only in 12 patients wherein the screw trajectory was determined to not be infected or destroyed via preoperative computed tomography (CT).

**Figure 1:**
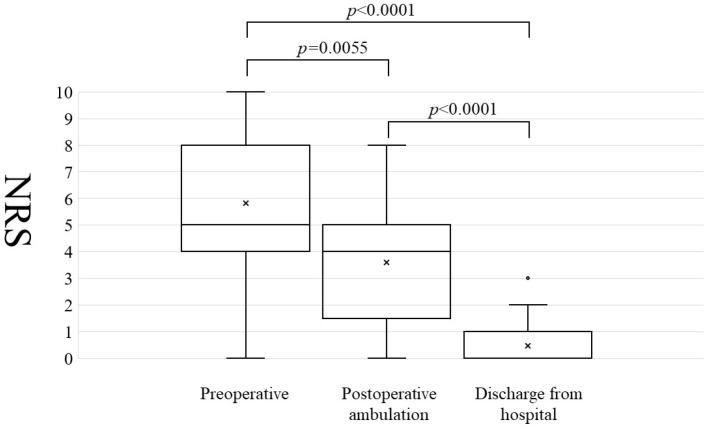
Numerical rating scale trends for lower back pain. Numerical Rating Scale: NRS trends for lower back pain before surgery, at the time of postoperative ambulation, and at discharge. NRS was significantly improved at the time of postoperative ambulation compared with that before surgery (5.8 vs. 3.6. *p* = 0.0055. Wilcoxon signed-rank test).

The mean duration of postoperative intravenous antibiotic administration was 24.8 days. Wound dehiscence occurred in three patients, screw back-out occurred in one, and proximal adjacent vertebral fracture occurred in one. The mean lower back pain NRS at preoperative/postoperative ambulation/discharge from hospital was 5.8/3.6/0.5, respectively (Figure 1). The lower back pain NRS significantly improved during postoperative ambulation compared with the preoperative value (*p* = 0.006). The average number of postoperative days required before ambulation was 2.7 days. The mean time required for the normalization of the C-reactive protein concentration following surgery was 42.8 days.

**Figure 2:**
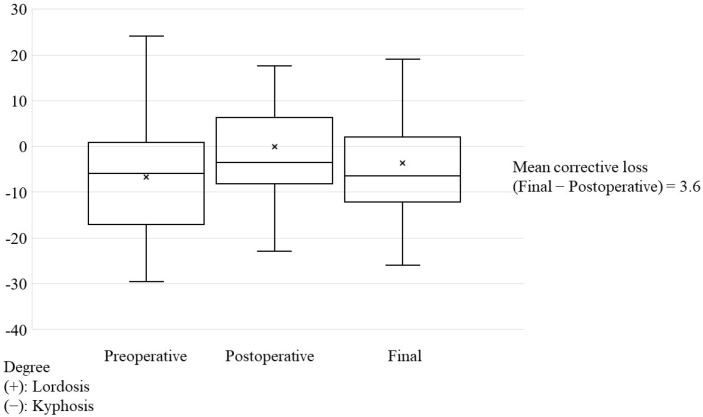
Progress of local kyphosis. Cobb angle was measured above and below the infected vertebrae. Surgery significantly improved local kyphosis by a mean of 6.6 degrees compared with that at preoperative conditions (*p* < 0.0001). At the last follow-up, the mean corrective loss was 3.6°, which was a better improvement than that before surgery (*p* = 0.023).

Local kyphosis averaged at 6.7°, 0.1°, and 3.7° at preoperative, postoperative, and last follow-up, respectively, (Figure 2), and significantly improved postoperatively compared with at preoperative conditions (*p* < 0.0001). At the last follow-up, the mean corrective loss was 3.6°, which was significantly more than that at preoperative conditions (*p* = 0.023).

**Figure 3:**
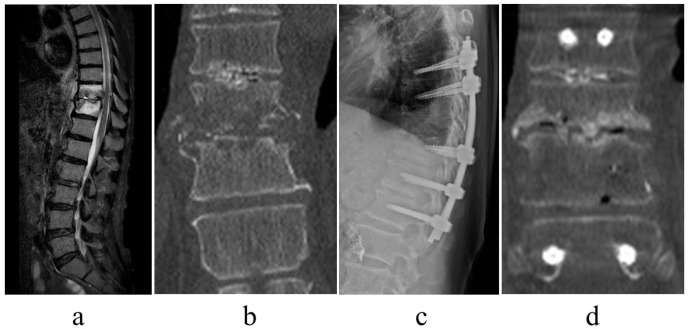
Images of a representative case of osteosclerosis of the endplate. A 71-year-old man, dialysis because of chronic renal failure, T10/11 pyogenic spondylitis. Short tau inversion recovery of magnetic resonance imaging revealed high signal changes in the vertebral body ((**a**), sagittal view), and computed tomography (CT) demonstrated significant bone destruction of the endplate((**b**), coronal view). The patient experienced persistent pain and underwent the posterior fixation of T8–L1 with percutaneous pedicle screw ((**c**), plain radiograph of thoracolumbar lateral view). One year postoperatively, the vertebral body was slightly crushed, and the endplate was osteosclerotic ((**d**), coronal view of CT). The patient exhibited improvement without recurrence of infection.

**Figure 4:**
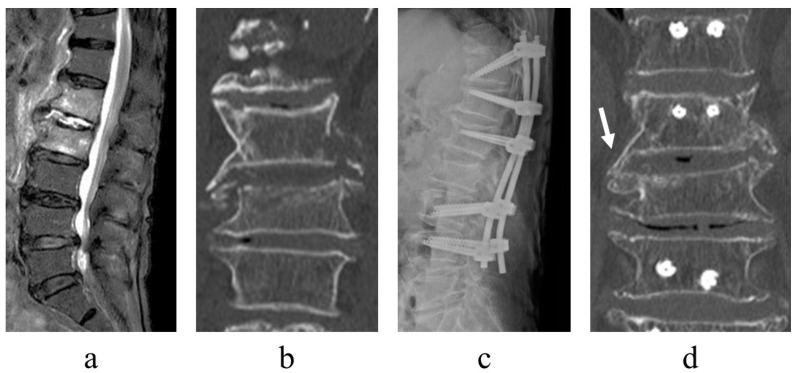
Image of a representative case of postoperative lateral bony bridge. An 87-year-old woman, L1/2 pyogenic spondylitis. Short tau inversion recovery on magnetic resonance imaging revealed signal changes in the vertebral body (**a**), and computed tomography (CT) demonstrated bone destruction and resorption of the L2 vertebral cranial endplate ((**b**), coronal view). The patient experienced persistent back pain and underwent posterior fixation of T11–L4 with percutaneous pedicle screw ((**c**), plain radiograph of thoracolumbar lateral view). One year postoperatively, a lateral bony bridge was obtained between the lateral L1 and L2 vertebrae ((**d**), CT coronal vie9w, arrow).

There was no recurrence of infection. Following surgery, there were cases of bone formation within the osteolytic lesion in destroyed vertebral endplates (Figure 3) alongside cases of lateral bony bridge following minimally invasive posterior fixation (Figure 4).

**Figure 5:**
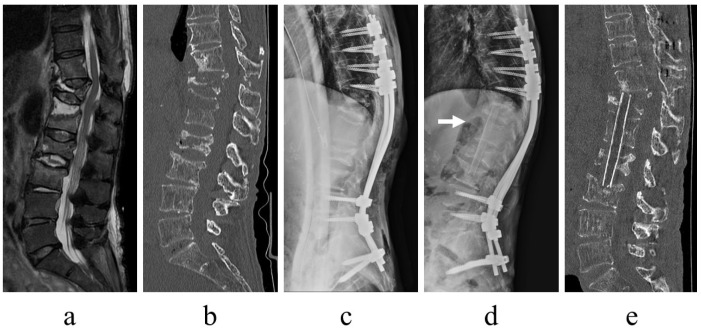
Images of a representative case with additional anterior surgery. An 81-year-old woman, T11/12/L1, L2/3 pyogenic spondylitis. Fat-saturated T2-weighted magnetic resonance imaging revealed signal changes from T11 to L3 vertebrae and disk (**a**), and computed tomography (CT) demonstrated large bone destruction at T12–L2, including preexisting vertebral fractures ((**b**), sagittal view). The patient experienced persistent back pain and underwent posterior fixation of T8–S2-alar-iliac ((**c**), plain radiograph of thoracolumbar lateral view). The infection persisted postoperatively, and an anterior debridement and autogenous bone grafting of the fibula were additionally performed ((**d**), plain radiograph of thoracolumbar lateral view, arrow, fibula; (**e**), CT sagittal view). Following additional anterior surgery, the infection was quickly controlled.

Six patients required additional surgery. Minimally invasive posterior fixation alone did not improve C-reactive protein concentration even ~2 weeks postoperatively, and infection control was considered insufficient in three patients (9.6%) who required anterior lesion debridement and autogenous bone grafting with the fibula or iliac bone (Figure 5). One patient required additional percutaneous drainage owing to a residual epidural abscess, one patient required percutaneous vertebroplasty for a proximal adjacent vertebral fracture, and one patient with diffuse idiopathic skeletal hyperostosis required additional fixation extension for back-out of a screw on the proximal level at 2 weeks postoperatively. Thus, 4 of the 31 patients (13%) required additional surgeries for infection control.

## 4. Discussion

The proportion of patients with pyogenic spondylitis has increased in recent years owing to the increase in the older population [14]. As less invasive surgical procedures have recently become increasingly popularized, less invasive surgeries for pyogenic spondylitis, including PPS, minimally invasive anterior surgery, and percutaneous endoscopic debridement, have been reported [2,3,4,5,6,7,11,12,13,15]. Minimally invasive posterior fixation without anterior debridement and autogenous bone grafting is potentially less invasive than conventional anterior debridement and, consequently, more appropriate for older patients and those with compromised immune systems [11,12,13]. Herein, the mean age of the patients was 73.3 years, with 71% patients >70 years in age, and several patients underwent treatment without major complications, thereby indicating that minimally invasive posterior fixation is also feasible for older patients.

The three goals of pyogenic spondylitis treatment include the following:

(1) Infection control. With minimally invasive posterior fixation and sensitive antibiotics, the inflammatory response normalized at an average of 42.8 days postoperatively. Previous reports stated that the period of normalization of the C-reactive protein concentration ranged from <1 month to 3 months; however, it was 42.8 days in this series [5,16]. In numerous patients, infection control was achieved without requiring additional surgery, thus implying that this technique is useful for infection control.
(2) Early ambulation. Less invasive procedures and pain reduction aid early ambulation. Herein, surgery significantly reduced pain (NRS for preoperative lower back pain was 5.9, which improved to 3.6 at the postoperative period of ambulation) and induced early ambulation (patients were able to ambulate at an average of 2.7 days postoperatively). For pain relief and time to ambulation, the results were comparable with those of previous studies [17,18]. Even in older patients, the minimally invasive procedure may enable early ambulation.
(3) Alignment maintenance. Local kyphosis, which is exacerbated by the destruction of the disk or vertebral body due to infection, is a measure of alignment maintenance. Minimally invasive posterior fixation improved local kyphosis by 6.6 degrees, with a postoperative correction loss of 3.6 degrees, which is comparable with results reported in previous studies (range: 3.4–6.8 degrees) [11,15,16]. These findings suggest that the range of fixation and fixation strength in this series were sufficient to maintain local alignment.

A major issue in minimally invasive posterior fixation for pyogenic spondylitis is determining which patients require anterior debridement and autogenous bone grafting. Herein, there were four cases (13%) of poor infection control, with three (9.6%) requiring anterior surgery, which is comparable with the findings of previous reports (range: 2.2–6.7% cases) [5,10].

The previously reported conditions for the successful treatment of pyogenic spondylitis with posterior fixation alone included the destruction of one-third or less of the vertebral body, removal of giant abscesses, insertion of pedicle screws into infected vertebrae, and the lesion being in an early stage [5,16,18,19]. Conversely, certain studies have reported that anterior bone grafting is necessary for large bone defects, such as when the vertebral body destruction exceeds two-thirds [15]. Herein, two of the three patients who had undergone additional anterior surgery exhibited significant bone destruction. The results of this series indicate that treatment with minimally invasive posterior fixation alone is difficult in patients with significant bone destruction where posterior fixation is not expected to provide sufficient local stability [7,17,19,20]. More case studies are warranted to identify cases where minimally invasive posterior fixation is contraindicated.

This study has some limitations. Despite the fact that this is a multicenter study, the patients are from a limited geographic area and restricted to an aging country. Therefore, the results of this study cannot be generalized to all patients. As a selection bias, we excluded patients that required planned debridement of infected lesions and bone grafting, thereby underestimating the proportion of patients requiring additional surgery. Another limitation of the current study is the short follow-up period. Mid- or long-term follow-up is warranted to determine the long-term maintenance of alignment and the feasibility of implant removal.

## 5. Conclusions

Minimally invasive posterior fixation constitutes a useful treatment option for thoracolumbar pyogenic spondylitis resistant to conservative treatment. The fact that posterior surgery alone cannot control infection in 13% of patients poses a future challenge in this treatment modality.

## Figures and Tables

**Table 1:** Patient characteristics.

	*n*
All patients	31
Sex	
Men	23
Women	8
Age (years)	73.3
Preoperative conservative treatment period (days)	52.7
Postoperative period of follow-up (months)	13.7
Site of involvement	
Lumbar	16
Thoracic	9
Thoracolumbar	4
Lumbosacral	2
Involved intervertebral	
1	25
2	4
3	1
4	1
Paravertebral abscess	17
Epidural abscess	13
Iliopsoas abscess	10
Comorbidity	
Diabetes	6
Cancer	6
Abdominal infection	5
Liver cirrhosis	1
Hemodialysis caused by chronic renal failure	1
Daily steroid use	1

**Table 2:** Causative organisms.

Organism	*n*
*Escherichia coli*	6
*Streptococcus* species	6
MSSA	2
MRSA	1
*Klebsiella pneumoniae*	1
*Peptostreptococcus* species	1
*Gemella haemolysans*	1
*Bacteroides fragilis*	1
None identified	12

MSSA: methicillin-susceptible *Staphylococcus aureus*, MRSA: methicillin-resistant *Staphylococcus aureus*.

## Data Availability

The datasets generated during and analyzed during the present study are available from the corresponding author on reasonable request.
